# Enhancing the Flexibility and Hydrophilicity of PLA via Polymer Blends: Electrospinning vs. Solvent Casting

**DOI:** 10.3390/polym17060800

**Published:** 2025-03-18

**Authors:** Qi-Hong Weng, Ming-Hsien Hu, Ji-Feng Wang, Jin-Jia Hu

**Affiliations:** 1 Department of Mechanical Engineering, National Yang Ming Chiao Tung University, Hsinchu 300, Taiwan; a0953661618@gmail.com (Q.-H.W.); www.806640420@gmail.com (J.-F.W.); 2 Department of Post-Baccalaureate Medicine, National Chung Hsing University, Taichung 402, Taiwan; minghsienhu@nchu.edu.tw; 3 Orthopedic Department, Showchwan Memorial Hospital, Changhua 500, Taiwan

**Keywords:** solvent blending, blend electrospinning, solvent mixture, evaporation-induced phase separation, non-solvent-induced phase separation, mechanical properties, crystallinity

## Abstract

Polylactic acid (PLA) is a biodegradable polymer with high tensile strength, high stiffness, and biocompatibility, but its brittleness and hydrophobicity limit its applications. This study aims to address these limitations by blending PLA with polycaprolactone (PCL) to enhance flexibility and with polyethylene oxide (PEO) to improve hydrophilicity. Unlike conventional approaches where PEO serves as a plasticizer, this study investigated PEO as a major blend component. Electrospinning and solvent casting, which differ in their solvent evaporation rates, were employed to fabricate thin films of neat PLA and PLA blends to examine their influence on mechanical and surface properties. Polymer solutions were prepared using a dichloromethane (DCM)/dimethylformamide (DMF) mixture known to enhance the electrospinning process. Fourier transform infrared spectroscopy (FTIR), X-ray diffraction (XRD), and differential scanning calorimetry (DSC) were used to investigate crystallinity of polymers and their interactions, while scanning electron microscopy (SEM) and atomic force microscopy (AFM) provided insights into phase separation and fiber morphology. Uniaxial tensile testing and water contact angle measurements were conducted to evaluate mechanical properties and surface properties, respectively. The results showed that electrospun PLA films exhibited higher elongation at break and ultimate strength but lower Young’s modulus than solvent-cast PLA films. Electrospun films of PLA/PCL blends demonstrated improved elongation at break while retaining Young’s modulus comparable to that of electrospun PLA films, unlike their solvent-cast counterparts. In contrast, PLA/PEO blends exhibited enhanced hydrophilicity in both processing methods but showed a marked reduction in mechanical properties. In summary, electrospun films consistently outperformed solvent-cast films in terms of flexibility and mechanical integrity, primarily due to their fibrous structure, suppressed phase separation, and reduced crystallinity. This study uniquely demonstrates that electrospinning enables the fabrication of phase-separated PLA/PEO blends with mechanical integrity despite PEO’s inherent immiscibility with PLA and incompatibility in the solvent mixture. Furthermore, electrospinning proves to be an effective processing method for producing PLA blend films with enhanced flexibility and hydrophilicity without the need for plasticizers or compatibilizers.

## 1. Introduction

The increasing environmental concerns over non-biodegradable plastics have accelerated the development of biodegradable alternatives from renewable resources. These biodegradable polymers, whether naturally derived or synthetically produced, are gaining widespread use in diverse applications, such as textiles [1], food packaging [2], tissue engineering [3], drug delivery [4], and 3D printing [5]. Their appeal is primarily due to their biocompatibility and reduced environmental impact.

Polylactic acid (PLA), a biodegradable thermoplastic polymer synthesized from renewable resources such as corn and sugarcane, is among the most widely used biopolymers due to its good mechanical strength and biocompatibility. However, its brittleness and poor ductility at room temperature significantly limit its applications. While plasticizers have traditionally been used to enhance the flexibility of PLA [6], they can negatively affect biocompatibility and increase production costs. Therefore, alternative strategies are needed to mitigate its brittleness while maintaining its desirable properties.

Polymer blending is an effective approach to modifying the mechanical properties of PLA without the complexity of synthesizing new copolymers, which requires precise control over monomer composition and reaction conditions [7]. By blending PLA with other polymers, complementary properties can be achieved, resulting in improved flexibility, toughness, and processability. This study investigated the use of polycaprolactone (PCL) and polyethylene oxide (PEO) as blend components for PLA. PCL, a biodegradable polyester, is known for its low melting point and excellent flexibility, while PEO is a hydrophilic polymer with good biocompatibility and mechanical adaptability. These polymers were selected to address PLA’s inherent limitations; PCL enhances flexibility and toughness, whereas PEO improves hydrophilicity and overall processability [8,9,10,11]. Notably, unlike most previous studies in which PEO serves as a minor plasticizer, this study explored PEO as a major blend component.

Beyond material selection, processing methods play a crucial role in determining the final properties of polymer blends. This study employs two fabrication techniques, electrospinning and solvent casting, to investigate their influence on the structural and mechanical properties of PLA-based blends.

Electrospinning is a versatile technique for producing ultrafine fibers at the nanometer to micrometer scale by applying a high-voltage electric field to a charged polymer solution. As the charged jet is ejected from the spinneret and accelerates toward a grounded collector, instabilities such as whipping and bending promote extensive elongation and thinning of the fiber. Concurrent solvent evaporation solidifies the polymer, forming non-woven fibrous membranes with high surface area-to-volume ratios and tunable fiber diameter, morphologies, and orientations [12,13]. Previous studies have demonstrated that electrospinning enhances polymer flexibility, elongation, and mechanical strength [14,15], making it a promising technique for modifying PLA-based materials.

Solvent casting, a widely used film fabrication method, involves dissolving a polymer in a suitable solvent and casting the solution onto a flat surface [16]. As the solvent gradually evaporates, the polymer precipitates, resulting in a solid, uniform film. This method is valued for its simplicity and ability to control film crystallinity by adjusting solvent evaporation rate [5,17].

The objective of this study is to systematically evaluate how different processing methods—electrospinning and solvent casting—affect the morphology, crystallinity, phase behavior, and mechanical properties of PLA and its blends with PCL and PEO. A comprehensive set of analytical techniques was employed to elucidate structural and mechanical characteristics of these materials. Scanning electron microscopy (SEM) was used to examine the surface morphology and fracture surfaces of the films, while Fourier transform infrared spectroscopy (FTIR), X-ray diffraction (XRD), and differential scanning calorimetry (DSC) provided insights into crystallinity of polymers and their interactions. Atomic force microscopy (AFM) enabled nanoscale characterization of crystalline morphology and phase-separated domains on individual fibers. In addition, uniaxial tensile testing evaluated mechanical properties, correlating mechanical performance with structural and thermal characteristics. Finally, water contact angle measurements assessed the surface hydrophilicity of the films.

By examining these structure–property relationship, this study provides insights into how polymer blending and processing methods influence the mechanical and surface properties of PLA-based materials. The findings enhance the understanding of material performance, guiding the development of optimized PLA-based films for diverse applications, particularly in biodegradable food packaging, biomedical materials such as wound dressings, and sustainable agricultural mulch films.

## 2. Materials and Methods

### 2.1. Materials

Polylactic acid (PLA) (Ingeo 3001D; D-lactide content: 1.4%) was obtained from NatureWorks (Minnetonka, MN, USA). Polycaprolactone (PCL) (Mn = 80,000) and polyethylene oxide (PEO) (Mn = 100,000) were purchased from Sigma-Aldrich (St. Louis, MO, USA). Both dichloromethane (DCM) and dimethylformamide (DMF) were of 99% purity and were obtained from Macron (Center Valley, PA, USA).

### 2.2. Electrospinning Setup

The electrospinning setup consisted of a syringe pump (KDS-210, KD Scientific, Holliston, MA, USA), a high-voltage power supply (AU-30P1-LC, Matsusada Precision, Kusatsu, Japan), and a grounded rotating drum (diameter 102 mm). A crank-slider mechanism was designed and employed to provide the reciprocating motion of the spinneret, ensuring a uniform film thickness. The voltage difference between the positively charged spinneret and the grounded collector was controlled by the high-voltage power supply. The flow rate was precisely controlled by the syringe pump, and the drum’s rotational speed was adjusted using the DC power supply. The working distance was adjustable.

### 2.3. Preparation of Electrospun Films

**Figure 1:**
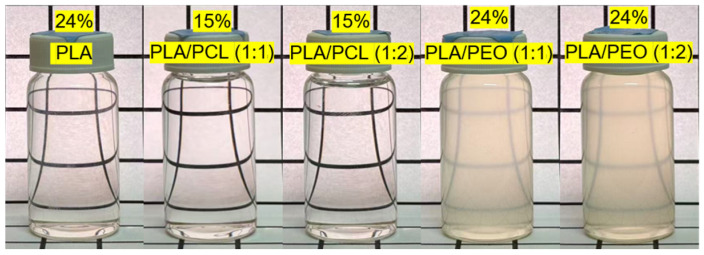
Photographs of the polymer solutions of neat PLA, PLA/PCL (1:1 and 1:2) blends, and PLA/PEO (1:1 and 1:2) blends.

Polymer solutions for electrospinning were prepared by dissolving the polymers in a 7:3 volumetric mixture of DCM and DMF, with heating at 50 °C to facilitate dissolution. This solvent mixture has proven effective for enhancing the electrospinning process [18], and we have successfully used it to fabricate electrospun PLA or PCL films with satisfactory results [3,19]. DCM, with its high volatility, promotes rapid fiber solidification and reduces bead defects, while DMF, due to its low volatility and higher conductivity, stabilizes the Taylor cone, which is crucial for the formation of consistent fibers. A 24 wt% solution was used to electrospin neat PLA. Electrospinning parameters were optimized to achieve uniform fiber morphology without bead formation. A voltage of 13–16 kV was applied to maintain a stable Taylor cone, while a flow rate of 4.8 mL/h ensured continuous fiber formation. The working distance of 15 cm between the spinneret and the collector was set to 15 cm, allowing sufficient time for solvent evaporation before fiber deposition. The electrospun fibers were collected for 90 min to form a uniform film. PLA was also solvent blended with PCL or PEO at weight ratios of 1:1 and 1:2. Note that the total polymer concentration was 15 wt% for the PLA/PCL blends and 24 wt% for the PLA/PEO blends. These concentrations ensured proper chain entanglement, preventing electrospraying or bead formation at lower concentrations and clogging at higher concentrations. Figure 1 shows the polymer solutions used in this study. With the exception of the turbid but macroscopically homogeneous PLA/PEO blend solutions, all other solutions were transparent and homogeneous, indicating that the solutes were fully dissolved at the specified concentrations in the DCM/DMF mixture. Table 1 summarizes the solubility of PLA, PCL, and PEO in DCM, DMF, and the DCM/DMF mixture. PEO appeared insoluble in DCM, DMF, and the solvent mixture. When a small amount of PEO was introduced into DCM, DMF, or their mixture, it swelled, possibly due to solvent absorption, with no clear evidence of dissolution. Consequently, the poor solubility of PEO in the DCM/DMF solvent system led to phase separation in the PLA/PEO blend solutions, likely resulting in a PEO-rich phase dispersion within the PLA solution. We also observed macroscopic phase separation in a 2:1 PLA/PCL blend solution in a preliminary investigation; therefore, this ratio was excluded from this study. For the electrospinning of the PLA blends, the voltage was adjusted to 14–18 kV to maintain a stable Taylor cone, while the other parameters remained the same as those used for neat PLA.

The electrospun films of neat PLA and PLA/PCL blends had thicknesses of approximately 0.2–0.3 mm, whereas those of PLA/PEO blends ranged from 0.3 to 0.5 mm. These measurements were obtained using a custom thickness gauge consisting of a digital micrometer head (Mitutoyo, Kanagqwa, Japan) and a strain-gauged cantilever for contact force measurement [3].

### 2.4. Preparation of Solvent-Cast Films

Solvent-cast films were prepared using the same polymer solutions as those employed in electrospinning for comparison. The solution was poured into a Teflon dish and covered with a size-matched glass funnel to suppress evaporation [15]. The solvent was allowed to evaporate at room temperature for 24 h, followed by an additional 24 h in a vacuum chamber to remove any residual solvents. The solvent-cast films of neat PLA had thicknesses of approximately 0.2 mm, whereas those of the PLA blends were around 0.1 mm. The thickness measurements were conducted using the custom thickness gauge.

### 2.5. Characterization of the Films

#### 2.5.1. Scanning Electron Microscopy

Scanning electron microscopy (SEM) was used to examine the microstructure of the top surface and fracture surface of the films. In PLA/PEO blend films, both electrospun and solvent-cast, additional SEM analysis was performed after water washing to selectively remove the PEO component. Energy-dispersive X-ray spectroscopy (EDS) was employed to analyze the elemental composition of phase-separated domains, allowing for the identification of the polymer (PLA, PCL, or PEO) based on known polymer compositions in the specimen. All specimens were Pt-coated before imaging. SEM and EDS analyses were conducted using a field-emission scanning electron microscope (FE-SEM SU-8010, Hitachi, Tokyo, Japan).

#### 2.5.2. Fourier Transform Infrared Spectroscopy (FTIR)

FTIR spectroscopy was employed to investigate potential molecular interactions between PLA and the blended polymers (PCL and PEO) and to assess differences in polymer chain organization between electrospun and solvent-cast films. FTIR spectra were recorded using a Thermo Scientific Nicolet 6700 FTIR spectrometer equipped with an ATR module. The parameters were set to 64 scans per minute, 2 cm^−1^ resolution, and a wavenumber range of 600–4000 cm^−1^.

#### 2.5.3. X-Ray Diffraction (XRD) Analysis

XRD analysis provided insights into the crystallinity of the films, enabling a comparison of how the rapid solvent evaporation in electrospinning versus the slower evaporation in solvent casting influenced the crystalline structure of PLA and its blends. XRD analysis was performed using a Bruker AXS GmbH D8 Advance X-ray diffractometer with Cu Kα radiation (1.5418 Å). The scanning range was 10–80°, with a step size of 0.025°/step and a counting time of 0.21 s/step.

#### 2.5.4. Differential Scanning Calorimetry (DSC)

Differential scanning calorimetry (DSC) was performed to analyze the thermal properties of the films, thereby helping to correlate thermal properties with phase separation and polymer compatibility. DSC measurements were conducted using a TA Instruments DSC Q200 equipped with an RCS cooling system. Samples (3–5 mg) were cooled to 0 °C and then heated to 200 °C at a rate of 10 °C/min. The melting temperature ($T_{m}$), cold crystallization temperature ($T_{cc}$), melting enthalpy (${∆H}_{m}$), and cold crystallization enthalpy (${∆H}_{cc}$) were recorded. Crystallinity was determined by calculating the ratio of the measured melting enthalpy, adjusted for cold crystallization enthalpy when applicable, to the enthalpy of fusion of a fully crystalline polymer. Specifically, the crystallinity degree for PLA, PCL, and PEO in the films were calculated using the following equations.

(1) $$X_{c,\;PLA}\left(\%\right)=\left[\frac{{∆H}_{m,\;PLA}-{∆H}_{cc,PLA}}{{∆H}_{m,\;PLA}^{0}W_{PLA}}\right]\times 100$$

(2) $$X_{c,PCL}\left(\%\right)=\left[\frac{{∆H}_{m,PCL}}{{∆H}_{m,PCL}^{0}W_{PCL}}\right]\times 100$$

(3) $$X_{c,PEO}\left(\%\right)=\left[\frac{{∆H}_{m,PEO}}{{∆H}_{m,PEO}^{0}W_{PEO}}\right]\times 100$$

where $W_{PLA}$, $W_{PCL}$, and $W_{PEO}$ represent the weight fractions of PLA, PCL, and PEO in the blends, respectively. The theoretical melting enthalpy for PLA, ${∆H}_{m,\;PLA}^{0}$, was assumed to be 93 J/g [20,21]; for PCL, ${∆H}_{m,\;PCL}^{0}$, it was assumed to be 139.5 J/g [22,23]; and for PEO, ${∆H}_{m,\;PEO}^{0}$, it was assumed to be 213 J/g [24]. For neat polymer, $W_{polymer}=$ 1.

#### 2.5.5. Atomic Force Microscopy (AFM)

Atomic force microscopy (AFM) was used to examine the topography and crystalline structure of individual electrospun fibers. Amplitude imaging captured surface morphology details, while phase imaging provided insights into molecular chain alignment and crystalline domains. AFM analysis was performed in tapping mode using an SPA-300HV AFM (SII Nanotechnology, Tokyo, Japan) with HI’RES-C18/CR-AU probes.

#### 2.5.6. Uniaxial Tensile Testing

**Figure 2:**
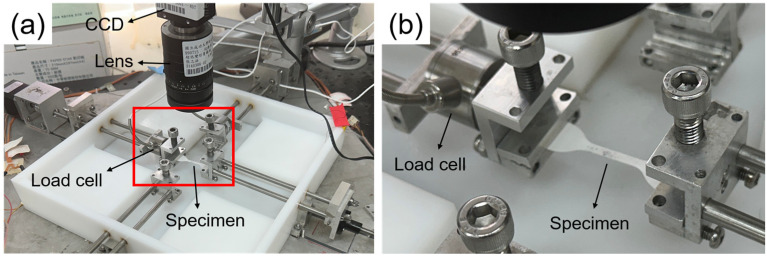
Photographs of the mechanical tester. The red rectangle in (**a**) highlights the region that is magnified in (**b**).

Uniaxial tensile testing was performed to evaluate the stiffness, tensile strength, and ductility of the films, directly linking processing-induced structural differences to material performance. Dog bone-shaped specimens were prepared by punching the electrospun and solvent-cast films using a miniature ASTM D412-C die (gauge length: 16.5 mm; width: 3 mm). Tensile tests were conducted with a custom-built mechanical tester equipped with a 1000-g load cell (WMCP-1000G, Interface, Scottsdale, AZ, USA), as shown in Figure 2. The specimens were stretched to failure at a strain rate of 0.0005 s^−1^. The stretch ratio in the loading direction was determined in real time by tracking the positions of twelve markers placed on the central surface of the specimen, relative to their initial, unloaded positions [25]. The stress in this central region was calculated from the tensile force recorded by the load cell and the deformed cross-sectional area, with the gauge width being 3 mm and the undeformed thickness of the specimen measured using the custom thickness gauge. Young’s modulus, ultimate strength, and elongation at break of the specimens were derived from the resulting stress–stretch curves as follows. Young’s modulus was defined as the initial slope of the stress–stretch curve, which remained relatively constant within the stretch ratio range of 1.00 to 1.01. The ultimate strength corresponded to the maximum stress, while the elongation at break was identified as the strain at which a sudden drop in stress occurred.

#### 2.5.7. Water Contact Angle Measurements

Water contact angle measurements were performed to assess the film surface wettability. Apparent water contact angles were measured using a sessile drop contact angle goniometer (FTA125, First Ten Angstroms, Portsmouth, VA, USA). Electrospun and solvent-cast films were cut into disc-shaped specimens (1 cm diameter) using a circular die. A 5-μL droplet of deionized water was deposited onto each specimen, and images were captured approximately two seconds after deposition to minimize the effects of water absorption and dynamic wetting. The average apparent contact angle for each group was determined from five specimens using the goniometer software (version 2.0).

### 2.6. Statistical Analysis

All data were reported as mean ± standard deviations. One-way ANOVA in conjunction with Tukey post hoc procedure was performed to access the difference between groups, with * indicating *p* < 0.05 and ** indicating *p* < 0.001.

## 3. Results and Discussion

### 3.1. Surface Morphology of the Electrospun Films

**Figure 3:**
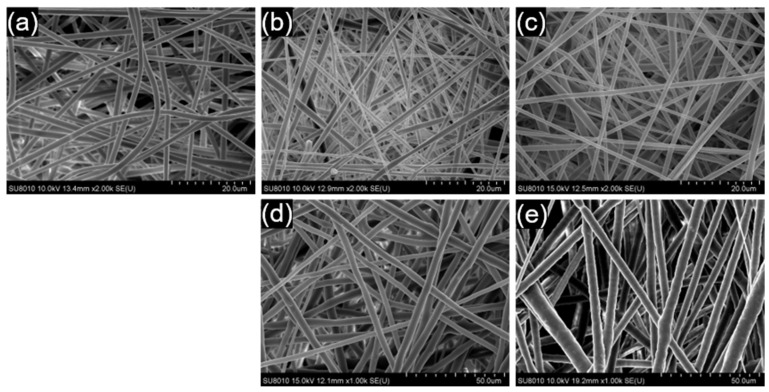
SEM images showing the top view of electrospun films: (**a**) neat PLA, (**b**) the PLA/PCL (1:1) blend, (**c**) the PLA/PCL (1:2) blend, (**d**) the PLA/PEO (1:1) blend, and (**e**) the PLA/PEO (1:2) blend.

Figure 3 illustrates top-view SEM images of the electrospun films of neat PLA, PLA/PCL blends, and PLA/PEO blends at different ratios. Neat PLA fibers exhibited smooth surfaces with an average diameter of 1.16 ± 0.23 µm. Incorporating PCL at the 1:1 ratio resulted in fibers with slightly more variation in diameter (0.95 ± 0.40 µm), whereas increasing the PCL content to the 1:2 ratio produced more uniform fibers with an average diameter of 0.93 ± 0.25 µm. The PLA/PEO (1:1) blend produced thicker fibers (3.11 ± 0.71 µm) with minor surface texturing, indicative of phase separation effects, while the PLA/PEO (1:2) blend generated the thickest fibers (4.18 ± 1.25 µm) with even rougher fiber surfaces, further reflecting the impact of phase separation on fiber morphology. The significantly larger diameters of PLA/PEO electrospun fibers are attributed to the poor solubility of PEO in the DCM/DMF mixture, which results in phase separation rather than the formation of a homogeneous polymer solution. In a homogeneous solution, the electrospinning jet undergoes smooth and uniform stretching, producing fine fibers. However, phase-separated PEO domains can disrupt this process, leading to uneven stretching and reduced jet thinning. In addition, localized viscosity variations within the jet may further resist elongation, contributing to the formation of thicker fibers.

### 3.2. Surface Morphology of the Solvent-Cast Films

**Figure 4:**
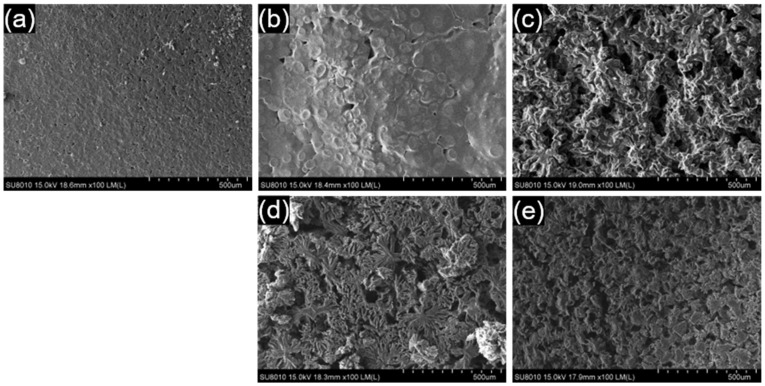
SEM images showing the top view of solvent-cast films: (**a**) neat PLA, (**b**) the PLA/PCL (1:1) blend, (**c**) the PLA/PCL (1:2) blend, (**d**) the PLA/PEO (1:1) blend, and (**e**) the PLA/PEO (1:2) blend.

Figure 4 shows top-view SEM images of the solvent-cast films, illustrating morphological variations influenced by polymer composition and blend ratios. The neat PLA film exhibited a relatively smooth surface with fine micro-textures and small pits. In contrast, the PLA/PCL (1:1) blend film exhibited circular or spherical domains, indicative of phase separation and the immiscibility of PLA and PCL. EDS analysis suggests that these domains are PLA-rich (~60% carbon, atomic %) and that the surrounding continuous matrix is PCL-rich (~70% carbon, atomic %), consistent with a previous study [26]. Voids were observed at the phase boundaries, likely resulting from poor interfacial adhesion. Increasing the PCL content to a 1:2 ratio led to a highly porous, interconnected surface, with PCL forming the continuous phase and PLA appearing as dispersed fragments within the matrix. In the PLA/PEO (1:1) blend, the surface displayed snowflake-like crystalline features. EDS analysis suggests that these features are PEO-rich (~65% carbon, atomic %). At the 1:2 PLA/PEO ratio, the morphology became more porous and irregular, with the snowflake-like crystalline structure becoming less distinct.

### 3.3. FTIR Analysis of the Electrospun and Solvent-Cast Films

**Figure 5:**
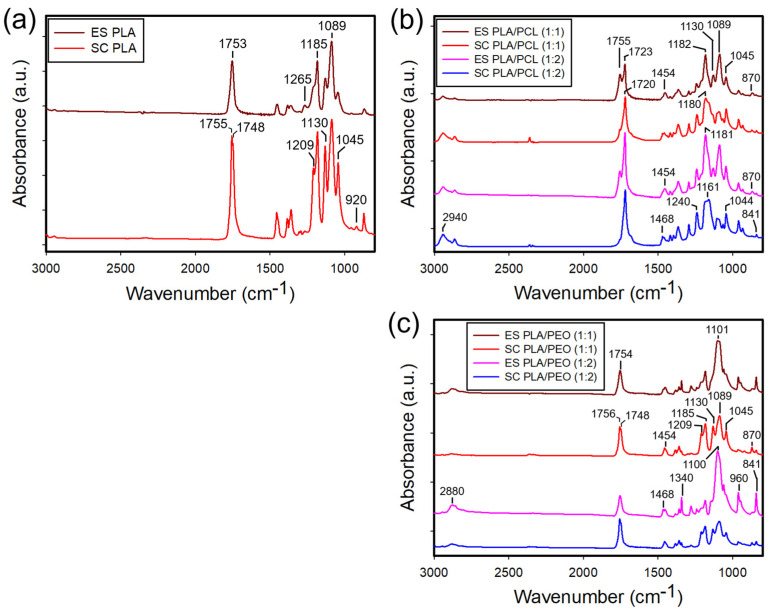
ATR-FTIR spectra of the electrospun and solvent-cast films of (**a**) neat PLA, (**b**) PLA/PCL (1:1 and 1:2) blends, and (**c**) PLA/PEO (1:1 and 1:2) blends.

Figure 5a shows the FTIR spectra of neat PLA films prepared by electrospinning and solvent casting. The solvent-cast films exhibited sharper and more intense absorption bands compared to the electrospun films, reflecting greater chain packing and crystalline order. Notably, the bands at 1045 cm^−1^ (C-CH_3_ stretching [27,28]) and 1130 cm^−1^ (CH_3_ rocking [27,28]) were more prominent in the solvent-cast films, relative to the band at 1750 cm^−1^ (C=O stretching). The 920 cm^−1^ band, linked to backbone C-C vibration and CH_3_ rocking [27,28], appeared exclusively in the solvent-cast films. This band has been attributed to the PLLA crystal phases [27,29] and its absence in the electrospun films suggests a lack of the crystal phases. Conversely, the 1265 cm^−1^ band (C-O-C stretching vibrations [27,28]) was stronger in the electrospun films than in the solvent-cast films, possibly due to differences in polymer chain packing.

Figure S1 shows the FTIR spectra of neat PCL films prepared by electrospinning and solvent casting. In contrast to PLA, the spectral features of PCL films were largely similar, except for a slightly more intense carbonyl stretching band at 1720 cm^−1^ in the solvent-cast films and minor peak shifts in the 1162–1186 cm^−1^ region. Characteristic absorption bands of PCL were identified at 2940 cm^−1^ (asymmetric stretching of CH_2_), 1720 cm^−1^ (C=O stretching), 1240 cm^−1^ (asymmetric stretching of C-O), and 1180 cm^−1^ (symmetric stretching of C-O), consistent with previous findings [30]. Figure 5b presents the FTIR spectra of PLA/PCL blend films prepared by electrospinning and solvent casting. Interestingly, the characteristic bands of PCL were more prominent in the solvent-cast films than in the electrospun films, with their intensity increasing with PCL content. Conversely, PLA absorption bands were more pronounced in the electrospun films. This observation suggests phase separation due to the immiscibility of PLA and PCL, where PCL, having a lower surface energy, preferentially migrates to the surface of solvent-cast films. However, rapid solvent evaporation during electrospinning limits this migration, resulting in greater PLA exposure. The observed variations in FTIR spectra between electrospun and solvent-cast films support varying extents of phase separation, reinforcing the limited miscibility of PLA and PCL in these blends.

Figure S1 also includes the FTIR spectrum of pristine PEO. Since PEO does not dissolve in the DCM/DMF mixture, electrospun films of neat PEO could not be fabricated using this solvent system. The characteristic absorption bands of PEO were identified at 2880 cm^−1^ (CH_2_ stretching), 1468 cm^−1^ (CH_2_ scissoring), 1340 cm^−1^ (CH_2_ wagging), 1100 cm^−1^ (C-O-C stretching), and 960 cm^−1^ (CH_2_ symmetric rocking), in agreement with previous findings [31]. Figure 5c shows the FTIR spectra of PLA/PEO blend films prepared by electrospinning and solvent casting. In this case, the PLA bands were more prominent in the solvent-cast films than in the electrospun films, regardless of the blend ratio. This suggests that PLA and PEO are immiscible, leading to phase separation. Similar to the PLA/PCL blends, phase separation in PLA/PEO blends appears to be governed by surface energy differences, with PLA preferentially migrating to the surface of solvent-cast films and PEO remaining more distributed in electrospun films due to rapid solvent evaporation. These observations further confirm that the phase behavior of the blends is highly dependent on processing conditions, with electrospinning reducing the extent of phase separation compared to solvent casting.

### 3.4. XRD Analysis of the Electrospun and Solvent-Cast Films

**Figure 6:**
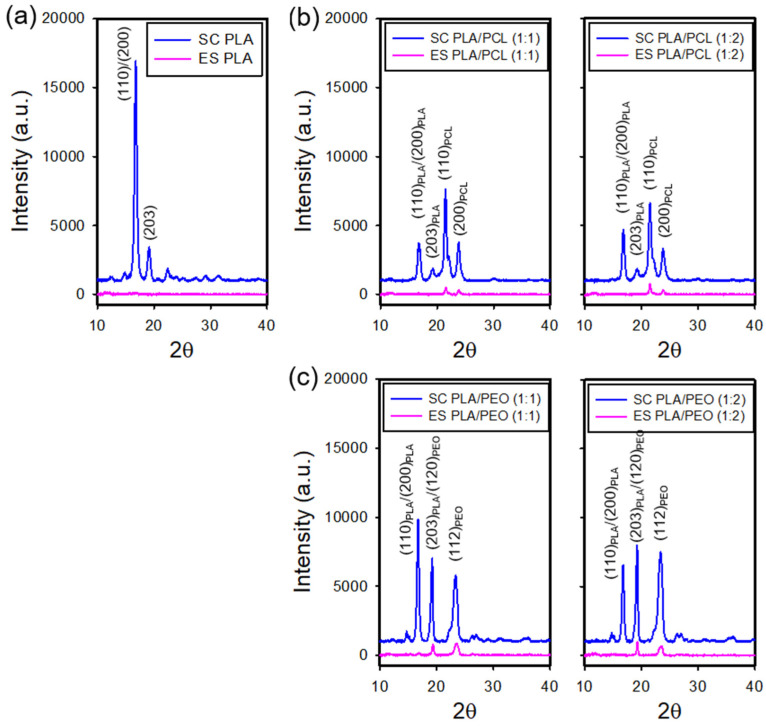
XRD patterns of the electrospun and solvent-cast films of (**a**) neat PLA, (**b**) PLA/PCL blends, and (**c**) PLA/PEO blends.

Figure 6a shows the X-ray diffraction (XRD) patterns of the electrospun and solvent-cast films of neat PLA. The solvent-cast films exhibited intense diffraction peaks at 2θ = 16.6° and 18.9°, corresponding to the (110)/(200) and (203) planes of PLA [32,33], respectively, indicating a higher degree of crystallinity. In contrast, the electrospun films showed a diffuse diffraction pattern with no well-defined peaks, suggesting smaller crystallite sizes or lower crystallinity. This may result from rapid solvent evaporation during electrospinning, which hinders molecular chain organization.

Figure S2 shows the XRD patterns of the electrospun and solvent-cast films of neat PCL and pristine PEO. The solvent-cast PCL films exhibited prominent diffraction peaks at 2θ = 21.3° and 23.7°, corresponding to the (110) and (200) planes of PCL [34], characteristic of its crystalline phase. In contrast, the electrospun PCL films displayed significantly reduced peak intensities at these positions, indicating a lower degree of crystallinity. Notably, while the characteristic peaks of PCL remained detectable in the electrospun films, the diffraction peaks of PLA were barely detectable in the electrospun PLA films, suggesting a greater suppression of PLA crystallization upon electrospinning. In addition, pristine PEO exhibited diffraction peaks at 2θ = 19.2° and 23.3°, corresponding to the (120) and (112) planes [35,36], confirming its crystalline nature.

Figure 6b and Figure 6c present the XRD patterns of the electrospun and solvent-cast PLA/PCL and PLA/PEO blends, respectively. In the solvent-cast films, diffraction peaks at 2θ = 16.9° and 18.9° corresponded to the (110)/(200) and (203) planes of PLA, while peaks at 2θ = 21.3° and 23.7° for PCL ((110) and (200) planes) and 2θ = 19.2° and 23.3° for PEO ((120) and (112) planes) indicate the presence of separate crystalline phases. This phase separation suggests that PLA was immiscible with both PCL and PEO in the crystalline state. Furthermore, the absence of peak shifts for PLA and PCL indicates minimal interactions between these polymers. In contrast, the electrospun films exhibited weakened PCL and PEO diffraction peaks with no discernible PLA peaks, indicating a largely amorphous structure. The reduced but retained diffraction peaks of PCL and PEO imply that phase separation persists in the electrospun blends, with PCL and PEO maintaining some crystalline domains while PLA remains amorphous. These results suggests that electrospinning suppressed crystallinity and may have enhanced polymer compatibility to some extent.

### 3.5. Thermal Properties of the Electrospun and Solvent-Cast Films

**Figure 7:**
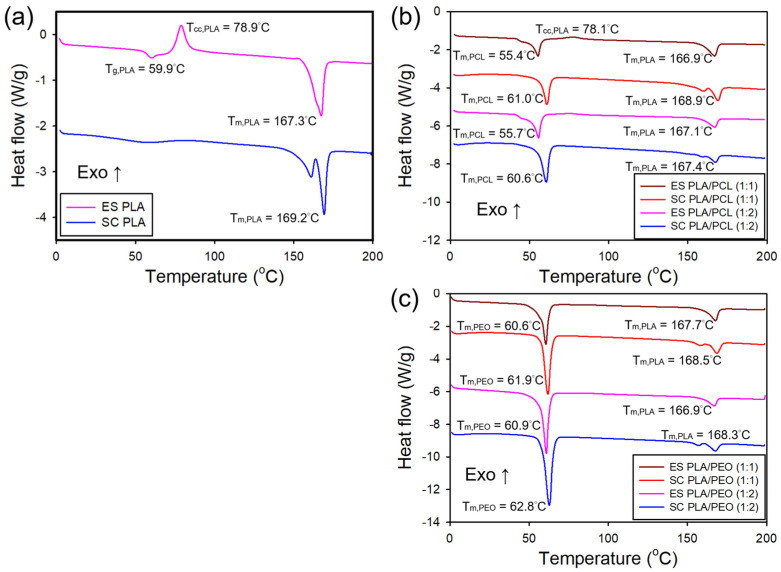
DSC thermograms of the electrospun and solvent-cast films of (**a**) neat PLA, (**b**) PLA/PCL blends, and (**c**) PLA/PEO blends.

Figure 7a shows the DSC curves for the electrospun and solvent-cast films of neat PLA. The electrospun films exhibited an endothermic transition around 57 °C corresponding to the glass transition temperature (*T*_g_), followed by an exothermic peak at 78 °C associated with cold crystallization temperature, and an endothermic peak at 167 °C corresponding to melting temperature (*T*_m_). The presence of a cold crystallization exotherm implies incomplete crystallization during electrospinning, with crystallization occurring upon heating above *T*_g_. In contrast, the solvent-cast films exhibited a broad endothermic transition near 57 °C, attributed to *T*_g_, and two endothermic melting peaks around 169 °C, with the absence of cold crystallization peak. The dual melting behavior in the solvent-cast films suggests the presence of multiple crystalline forms or a distribution of crystallite sizes, consistent with previous reports [37,38,39].

Figure 7b shows the DSC thermograms of the electrospun and solvent-cast films of PLA/PCL blends. The immiscibility of PLA and PCL was confirmed by the DSC curves of the blends, which exhibited two distinct melting peaks corresponding to PLA and PCL, consistent with a previous report [40]. This phase separation indicates that PLA and PCL did not form a homogeneous blend but rather remained as distinct domains. In the electrospun films, the DSC thermograms displayed an endothermic peak around 55 °C, corresponding to PCL melting, an exothermic peak near 78 °C, indicative of the cold crystallization of PLA, and an endothermic peak at approximately 167 °C, associated with PLA melting. The intensity of the exothermic peak related to PLA cold crystallization diminished with decreasing PLA content. In contrast, the DSC curves for the solvent-cast films exhibited an endothermic peak around 60 °C, corresponding to PCL melting (Figure S3a), along with dual endothermic peaks near 169 °C, attributed to the melting of PLA. Notably, the exothermic peak from PLA cold crystallization was absent in the solvent-cast films, indicating that PLA underwent more complete crystallization during solvent casting. In addition, the melting temperature of PCL was higher in the solvent-cast films compared to the electrospun films, likely due to increased crystallinity of the solvent-cast films. A similar trend was observed for the melting temperature of PLA, though the difference between the two processing methods was less pronounced.

Figure 7c shows the DSC thermograms of the electrospun and solvent-cast films of PLA/PEO blends. The presence of distinct melting peaks for PLA and PEO in both processing methods indicates that PLA and PEO are also immiscible. In the electrospun films, an endothermic peak at approximately 61 °C was observed, corresponding to the melting of PEO (Figure S3b), along with another endothermic peak around 167 °C, corresponding to the melting of PLA. In the solvent-cast films, an endothermic peak at approximately 62 °C and dual endothermic peaks at around 169 °C were detected, corresponding to the melting of PEO and PLA, respectively. Similar to PCL in the PLA/PCL blends, the melting temperature of PEO in the solvent-cast films was slightly higher than in the electrospun films. A similar trend was observed for the melting temperature of PLA.

Table 2 summarizes melting temperatures and degree of crystallinity for PLA, PCL, and PEO in various blends and processing methods. The pristine PEO is also included as a reference. The electrospun PLA films exhibited lower crystallinity than their solvent-cast counterparts. This reduction in crystallinity is primarily due to the rapid solvent evaporation and extreme tensile forces experienced during electrospinning. The rapid solidification freezes the polymer in a kinetically trapped, amorphous state before crystallization can occur. In addition, the high strain rates in the electrospinning process cause extensive chain stretching and alignment, limiting molecular mobility and preventing polymer chains from reorganizing into ordered crystalline structures. In contrast, solvent-cast films undergo slower solvent evaporation, allowing greater molecular mobility and facilitating crystal growth. Bognitzki et al. reported similar trends, supporting the role of processing conditions in governing crystallinity [41]. For PLA/PCL blends, the electrospun films exhibited lower PLA crystallinity than their solvent-cast counterparts. Notably, in the electrospun films, PLA crystallinity increased in the presence of PCL and further increased with higher PCL content. This can be attributed to the low *T*_g_ of PCL, which promotes PLA chain mobility, facilitating greater molecular rearrangement before solidification. In addition, the phase-separated interface arising from the immiscibility of PLA and PCL may act as a nucleation site for PLA crystallization. In contrast, PLA crystallinity in the solvent-cast films slightly decreased with increasing PCL content. This is likely due to PCL interfering with PLA crystal packing and contributing to the formation of larger phase-separated domains, which hinder uniform PLA crystallization. Similar trends were observed for PLA/PEO blends. The electrospun films had lower PLA crystallinity than the solvent-cast films. In the electrospun films, PLA crystallinity also increased with the addition of PEO. This increase may be due to the strong crystallization tendency of PEO, which can act as a nucleating agent for PLA, as well as its flexible nature, which enhances PLA chain mobility. In contrast, in solvent-cast films, PLA crystallinity slightly decreased as PEO content increased, likely due to PEO disrupting PLA crystal packing and modifying phase separation behavior.

### 3.6. Mechanical Properties of the Electrospun and Solvent-Cast Films

**Figure 8:**
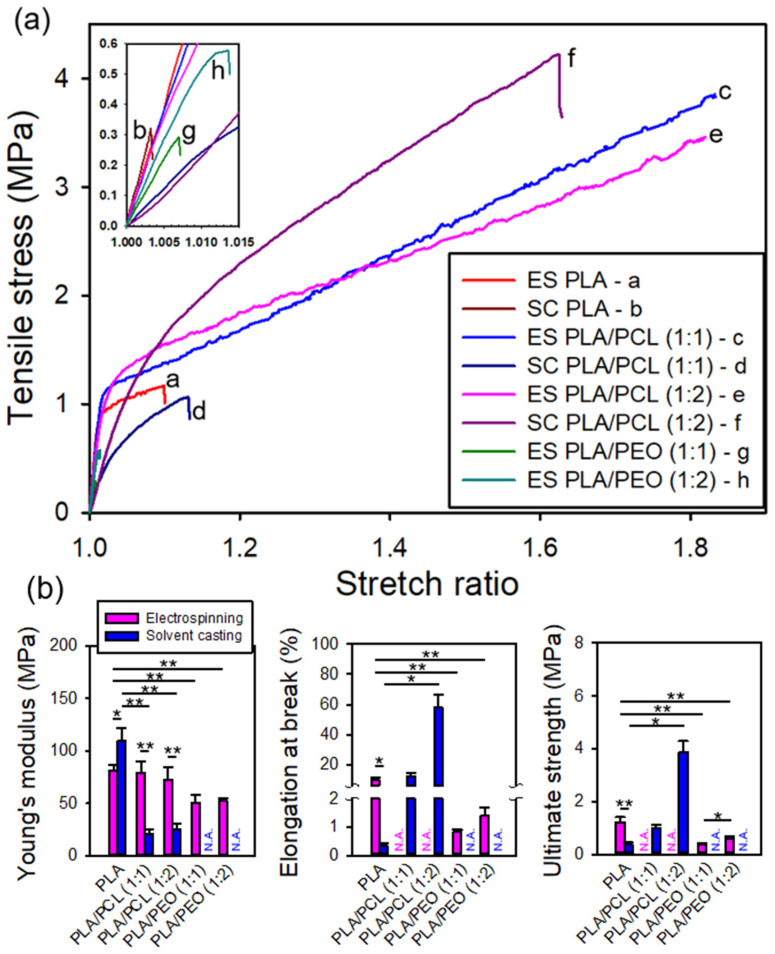
(**a**) Representative stress–stretch curves and (**b**) comparisons of the mechanical properties of the electrospun and solvent-cast films of neat PLA, PLA/PCL blends, and PLA/PEO blends. * indicating *p* < 0.05 and ** indicating *p* < 0.001.

Figure 8a presents the representative stress–stretch curves for the electrospun and solvent-cast films of neat PLA, PLA/PCL blends, and PLA/PEO blends. The electrospun PLA films exhibited clear yielding, lower stiffness, and greater toughness compared to the brittle solvent-cast PLA films, indicating improved flexibility. All PLA/PCL blend films and neat PCL films (Figure S4), whether electrospun or solvent-cast, displayed clear yielding behavior. However, the electrospun specimens, initially in sheet form, collapsed into a bundle at a stretch ratio of approximately 1.8, causing the surface markers to detach. This prevented the estimation of their elongation at break and, hence, ultimate strength. In addition, due to the extreme brittleness of the solvent-cast PLA/PEO blend films, intact specimens could not be obtained through die cutting, preventing mechanical testing. As a result, no data were available for solvent-cast PLA/PEO blend films.

**Figure 9:**
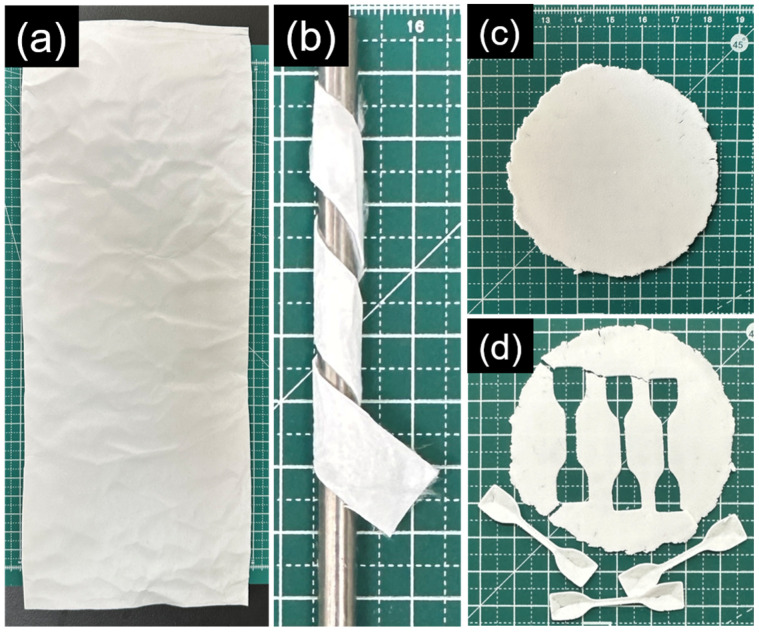
Photographs of the electrospun (**a**,**b**) and solvent-cast (**c**,**d**) films of PLA/PEO (1:1) blend.

Figure 8b presents statistical comparisons of the available mechanical properties of neat PLA, PLA/PCL blends, and PLA/PEO blends prepared by electrospinning and solvent casting. For neat PLA, the electrospun films exhibited lower Young’s modulus, higher ultimate strength, and greater elongation at break compared to their solvent-cast counterparts, with all differences being statistically significant. For PLA/PCL blends, electrospun films exhibited significantly higher Young’s modulus than their solvent-cast counterparts. Although the ultimate strength and elongation at break of the electrospun PLA/PCL films could not be determined, the elongation at break was presumed to exceed 80%, suggesting greater ductility than that of the solvent-cast films. The poor miscibility of PLA and PCL [42,43,44] likely led to phase separation during solvent casting, resulting in lower Young’s modulus. This observation is consistent with findings by Chen et al. [42], Murphy et al. [45], and Wei et al. [46], who reported that poor miscibility and weak interfacial adhesion between PLA and PCL reduce stiffness and strength in solvent-cast films. In contrast, significant phase separation due to poor polymer miscibility was not observed in electrospun films, likely because the rapid solvent evaporation during electrospinning kinetically traps the polymer chains in a mixed state. Previous studies have leveraged electrospinning to promote non-equilibrium miscibility in partially miscible polymer blends for various applications [47,48]. For PLA/PEO blends, both electrospinning and solvent casting exhibited significant mechanical weakening. However, electrospun PLA/PEO films exhibited a more homogeneous dispersion of PEO, preventing the formation of large brittle crystalline domains, which were evident in their solvent-cast counterparts. Figure 9 shows photographs of as-prepared PLA/PEO (1:1) blend films, with electrospun (Figure 9a) and solvent-cast (Figure 9c) samples. Figure 9b displays a ribbon of the electrospun film helically wrapped around a rod, highlighting its flexibility and conformability. Due to inherent phase separation in the PLA/PEO blend solution, the solvent-cast films were too brittle for intact specimen preparation via die-cutting for mechanical testing, as shown in Figure 9d.

Table 3 summarizes the mechanical properties of electrospun and solvent-cast PLA-based films, highlighting variations in Young’s modulus, ultimate strength, and elongation at break across different compositions and processing methods.

The mechanical properties of electrospun neat PLA, PLA/PCL blends, and PLA/PEO blends were statistically compared. Young’s modulus of electrospun PLA/PCL blends showed no significant difference from that of neat PLA, indicating that the addition of PCL did not notably affect film stiffness. However, PLA/PCL blends demonstrated considerably greater elongation at break compared to neat PLA, suggesting enhanced ductility. In contrast, the electrospun PLA/PEO blends exhibited significantly lower Young’s modulus, ultimate strength, and elongation at break than electrospun PLA films. This reduction in mechanical properties may be attributed to the phase-separated nature of the PLA/PEO blend solution. As previously noted, PEO did not dissolve in the DCM/DMF mixture, and when incorporated into the PLA solution, it formed a PEO-rich phase dispersion that remained electrospinnable. Intriguingly, the ultimate strength of the PLA/PEO blend (1:2) was significantly greater than that of the PLA/PEO blend (1:1).

Among the solvent-cast films, neat PLA exhibited the highest Young’s modulus but the lowest ultimate strength and elongation at break. The compromised mechanical properties of the solvent-cast PLA were likely attributed to the DCM/DMF mixture, as evidenced by the fracture surface of the films shown in the following section. The addition of PCL significantly reduced Young’s modulus of solvent-cast PLA/PCL films likely due to phase separation between PLA and PCL. The reduction in stiffness contrasts with their electrospun counterparts where phase separation was likely suppressed. Nevertheless, Young’s modulus, ultimate strength, and elongation at break all showed varying degrees of enhancement with increasing PCL content.

### 3.7. SEM Images of the Fracture Surface

**Figure 10:**
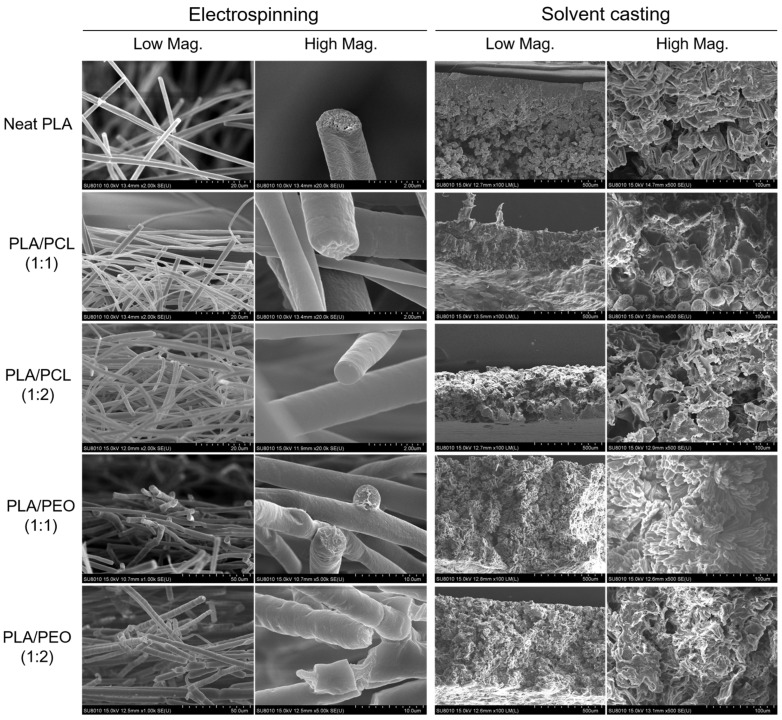
SEM images of the fracture surface of the electrospun and solvent-cast films of neat PLA, PLA/PCL blends, and PLA/PEO blends.

Figure 10 shows SEM images of the fracture surfaces of electrospun and solvent-cast films. To effectively identify phase-separated domains, images were captured at varying magnifications for each film type. The high-magnification SEM image of electrospun PLA fibers revealed a rough fiber surface with nanopores at the fractured end, likely resulting from a non-solvent-induced phase separation mechanism [49,50]. In contrast, SEM images of the solvent-cast PLA film exhibited a bilayer structure, with the air-facing top layer appearing more compact and the bottom layer more porous. This morphology likely results from the sequential evaporation of DCM and DMF, combined with the limited solubility of PLA in DMF, which influence solute migration and phase separation during solvent casting. Notably, when PLA was solvent-cast using DCM alone, the resulting film exhibited a compact fracture surface (Figure S5) but was extremely brittle.

While nanopores were absent at the fractured fiber ends, the surface and fractured ends of electrospun PLA/PCL blend fibers became smoother with increasing PCL content. In contrast, the solvent-cast PLA/PCL films exhibited significant phase separation. At the PLA/PCL ratio of 1:1, spherical structures were distributed throughout a continuous matrix on the fracture surface. EDS analysis confirmed that these spherical domains are PLA-rich, consistent with the spherical structures observed on the top surface of the same films. When the PLA/PCL ratio decreased to 1:2, the fracture surface became more irregular, likely due to increased deformation of the separated PLA and PCL phases prior to fracture. The noticeable reduction in the number of spherical structures also suggests that these domains correspond to PLA-rich phases.

The surface and fractured ends of electrospun PLA/PEO blend fibers appeared rough, with roughness increasing as PEO content increased. Notably, multiple instances of necking were observed along the fibers at the PLA/PEO ratio of 1:2, a phenomenon also reported in PHBV/PEO solution blends [51]. On the other hand, the fracture surface of the solvent-cast PLA/PEO film at the 1:1 ratio displayed snowflake-like structures, indicative of localized crystallization. This crystallization likely resulted from PEO forming microdomains within the PLA matrix during solvent casting. In addition, the rough, layered appearance of the fracture surface suggests a brittle fracture mechanism, likely driven by phase separation between PLA and PEO. At a higher PEO content (PLA/PEO ratio of 1:2), lamellar crystalline structures appeared on the fracture surface, reflecting increased PEO crystallinity. The porous and disrupted morphology further indicates poor interfacial adhesion between the PLA and PEO phases, likely due to the detachment of PEO-rich regions during fracture.

### 3.8. Selective Removal of PEO in PLA/PEO Blends

**Figure 11:**
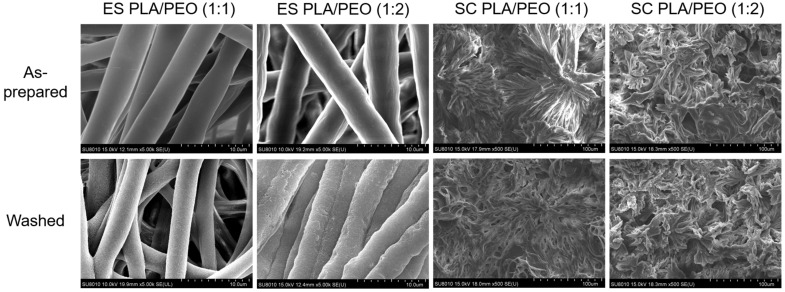
SEM images of the surface morphology of as-prepared electrospun and solvent-cast films of PLA/PEO blends and their water-washed counterparts.

PEO is water-soluble and can be selectively removed from PLA/PEO blend films through water washing. Comparing the films before and after water washing highlights the presence and distribution of the PEO phase. Figure 11 shows the SEM images of the electrospun and solvent-cast films of PLA/PEO blends before and after water washing, highlighting the distinct morphological changes associated with each processing method. Before water washing, the electrospun fibers exhibited relatively smooth surfaces, regardless of the PLA/PEO ratio. This observation suggests that the rapid evaporation during electrospinning suppressed large-scale phase separation, resulting in a more homogeneous distribution of PLA and PEO on the fiber surfaces. After water washing, the fiber surfaces became roughened, which was indicative of the selective removal of the water-soluble PEO phase. These morphological changes confirm that phase separation occurred at a microscopic scale, with PLA and PEO forming very fine domains within the fibers. In contrast, the solvent-cast films displayed rough, interconnected networks before water washing, indicative of macroscopic phase separation. The slower solvent evaporation during casting likely allowed sufficient time for PLA and PEO to segregate into larger, more distinct domains. After water washing, the solvent-cast films underwent dramatic morphological changes. The snowflake-like structures disappeared, confirming that these structures were PEO-rich. The surfaces exhibited extensive porosity and crystalline structures, particularly at the PLA/PEO ratio of 1:2. These findings further demonstrated that electrospinning suppressed phase separation, resulting in the fine dispersion of insoluble PEO within PLA and giving the appearance of miscibility.

### 3.9. AFM Characterization of Single Fiber Morphology

**Figure 12:**
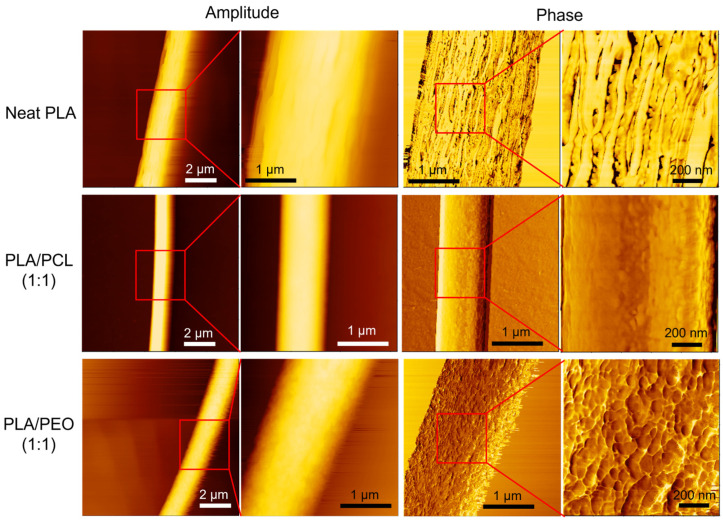
AFM images of a single electrospun PLA fiber. The red square highlights the region that is magnified in the adjacent image to the right.

AFM phase imaging highlights variations in material properties, such as stiffness, adhesion, and viscoelasticity, across the fiber surface, thereby providing insights into the nanoscale morphology and material heterogeneity of the fiber. Figure 12 shows AFM images of individual electrospun fibers, including a neat PLA fiber in the first row, a PLA/PCL blend fiber in the second row, and a PLA/PEO blend fiber in the third row. The leftmost amplitude image in the first row showed the overall fiber morphology of a neat PLA fiber, scanned over an area of 10 μm × 10 μm. The next two images are enlarged amplitude and phase images of the same region, with a scan area of 3 μm × 3 μm. The phase image showed brighter, elongated domains oriented parallel to the fiber axis. These stiffer domains may result from the alignment of PLA chains and the partial formation of crystalline structures during electrospinning. A further magnification of the phase image, with a scan area of 1 μm × 1 μm, clearly revealed that the elongated domains were aligned parallel to the fiber axis. This aligned fibrillar structure may explain the stiffness of the electrospun PLA films. The finding is consistent with a previous study, in which Lim et al. demonstrated that neat PCL electrospun fibers with aligned crystalline structures exhibited higher Young’s modulus and strength [52]. The leftmost amplitude image in the second row illustrates the overall fiber morphology of a PLA/PCL (1:1) blend fiber. The subsequent images show enlarged amplitude and phase images of the same region. The phase image revealed a rough surface texture with irregularly distributed domains. Upon further magnification, the phase image displayed finer, island-like domains, which are likely attributable to phase-separated regions (PLA-rich and PCL-rich regions) or crystalline structures. The third row illustrates a PLA/PEO blend (1:1) fiber. The low-magnification phase image revealed a rough fiber surface, characterized by longitudinal ridges and valleys, likely resulting from phase separation due to the immiscibility of PLA and PEO. The high-magnification phase image displayed distinct nanoscale pebble-like domains aligned along the fiber axis, further supporting this interpretation. These observations are consistent with a previous study by Goonoo et al., which reported similar morphological features in electrospun polymer blend fibers [53].

Figure S6 shows the AFM images of a single electrospun PCL fiber. Notably, needle-like structures were observed across the fiber surface in the phase image, which are likely crystalline structures. In contrast to the morphology shown in the phase images of a single PLA fiber, these structures were aligned more perpendicularly to the fiber axis.

The morphological results from SEM and AFM clearly illustrate fiber/film structure and nanoscale features associated with phase separation. To facilitate comparisons of blending composition and processing methods, these findings are summarized in Table S1.

### 3.10. Water Contact Angle Measurements of the PLA Films

**Figure 13:**
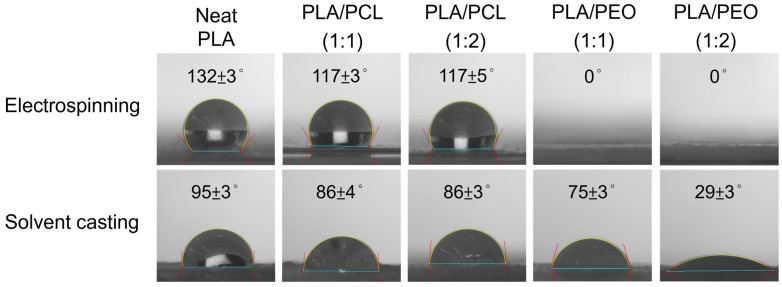
Representative photomicrographs of the water contact angles of the electrospun and solvent-cast films of neat PLA, PLA/PCL blends, and PLA/PEO blends.

Figure 13 shows apparent water contact angles of the electrospun and solvent-cast films of neat PLA, PLA/PCL blends, and PLA/PEO blends. The electrospun films of neat PLA, neat PCL (Figure S7), and PLA/PCL blends exhibited significantly higher contact angles than their solvent-cast counterparts, indicating greater hydrophobicity. This can be explained by the Cassie-Baxter model, which suggests that the rougher surface of electrospun films traps more air pockets, thereby enhancing hydrophobicity and elevating the contact angle [54]. More recently, Liu proposed that the surface hydrophobicity of electrospun and solvent-cast films is governed by distinct mechanisms [55]. During electrospinning, the hydrophobic groups of the polymer reorient toward the relatively nonpolar air-facing surface, resulting in a hydrophobic fiber surface [56]. In contrast, during solvent-casting, the earlier evaporation of more volatile solvents increases the overall solvent polarity, resulting in a hydrophilic surface [57].

For electrospun films, the contact angles of the PLA/PCL blends were significantly lower than those of neat PLA, whereas no significant differences were found between these blends and neat PCL (Figure S7). For solvent-cast films, no significant differences in contact angles were found among PLA and these blends.

The addition of PEO markedly enhanced the hydrophilicity of both electrospun and solvent-cast films. The contact angles of the solvent-cast PLA/PEO blend films were significantly smaller than those of their PLA counterparts, with further reductions as the PEO content increased. Notably, for both blend ratios, the apparent water contact angles of the electrospun PLA/PEO films reached zero. In fact, water droplets were instantly adsorbed upon contact with the electrospun films. This phenomenon is likely attributable to the synergistic effects of the hydrophilic nature of PEO and the porous structure of electrospun fibers. The wettability of solid surfaces is influenced by both chemical composition and surface morphology [58]. Natu et al. reported that PCL/Lu (Lu stands for Lutrol F127, a hydrophilic copolymer) blend fibers were highly hydrophilic, with water contact angle below 20°, attributing this behavior to the preferential arrangement of Lu at the fiber surface [59]. Indeed, the ATR-FTIR spectra of electrospun PLA/PEO blend films exhibited more pronounced PEO absorption bands than those of solvent-cast films, suggesting a higher surface concentration of PEO in electrospun fibers.

### 3.11. Limitations and Future Perspectives

DSC, AFM, and XRD each provide unique insights into the structural and crystalline properties of electrospun and solvent-cast films. DSC, highly sensitive to crystallinity, detects both well-ordered and imperfect crystalline regions by measuring melting enthalpy. AFM phase imaging, with nanoscale resolution, reveals localized crystalline alignment on electrospun fiber surfaces. In contrast, XRD primarily detects long-range periodic structures, making it less effective at identifying small, strained, or highly defective crystallites in electrospun fibers. In addition, preferred orientation effects in electrospun fibers can cause anisotropic diffraction, further reducing peak visibility. These distinctions emphasize the importance of integrating multiple characterization techniques to achieve a comprehensive understanding of crystallinity and molecular organization.

For comparison, we used the same solvent mixture to dissolve neat PLA as well as PLA/PCL and PLA/PEO blends, in preparing both electrospun and solvent-cast films. During electrospinning, rapid solvent evaporation kinetically traps the polymer phases, suppressing phase coarsening and resulting in finer phase domains. However, solvent casting involves a much slower evaporation process, allowing the differences in solvent volatilities and polymer solubilities to play a role in phase separation. As DCM evaporates more quickly, the residual DMF becomes enriched in the drying film, altering the local solvent environment. Since PLA has limited solubility in DMF, PEO is insoluble, and PCL dissolves only to some extent in DMF, this solvent shift can induce non-solvent-induced phase separation, leading to premature precipitation of PEO and PLA while allowing PCL to remain more solvated. This phase-selective precipitation leads to uneven polymer distribution, increased heterogeneity, and an irregular microstructure in the final solvent-cast films, raising concerns about reproducibility. While phase separation poses challenges for solvent casting, electrospinning can mitigate this issue if the solution is processed immediately after preparation, particularly for PLA/PEO blends, before significant phase separation occurs. Rapid solvent evaporation during electrospinning prevents large-scale phase separation, improving reproducibility. Thus, although the DCM/DMF mixture is unsuitable for solvent casting due to PEO’s insolubility, it remains viable for electrospinning if used promptly.

While our study focuses on specific polymer-solvent compositions, phase diagrams of the individual constituents could provide valuable insights into phase behavior during electrospinning and solvent casting. Such diagrams help predict miscibility, solubility limits, and phase separation, all of which influence film morphology and properties. In polymer blends, the polymer ratio plays a crucial role in phase compatibility and structural uniformity. Although constructing phase diagrams is beyond the scope of this study, they remain invaluable for optimizing both polymer and solvent ratios to enhance film formation and reproducibility.

Byun et al. reported that the crystallinity of solvent-cast PLA films is strongly influenced by the solvent system, with higher boiling point solvents leading to increased crystallinity but also to greater brittleness [17]. Given that solvent evaporation rate and polymer solubility govern both non-solvent-induced phase separation and crystallization, the choice of solvent system can significantly impact the final film morphology and mechanical properties. It remains an open question whether these effects extend to electrospun fibers. As solvent systems have been shown to influence the morphology of electrospun fibers [60], further investigation into a broader range of solvent systems for electrospinning may provide insights for optimizing fiber structure and mechanical performance.

The flexibility of the electrospun films may, in part, be attributed to their porous structure. The solvent-cast films, in general, lack pores. Introducing comparable porosity into solvent-cast films using porogens or alternative methods could provide more suitable candidates for comparison.

This study provides fundamental insights into how electrospinning influences the mechanical and surface properties of neat PLA and its blends with PCL and PEO. Future research should focus on optimizing blend compositions and processing parameters to further enhance film performance. In addition, investigating the biocompatibility and degradation behavior of these films both *in vitro* and *in vivo* will be crucial for their biomedical applications.

## 4. Conclusions

This study highlights the significant impact of processing methods—electrospinning versus solvent casting—on the mechanical and surface properties of thin films from neat PLA, PLA/PCL blends, and PLA/PEO blends. For neat PLA, electrospinning yielded films with higher elongation at break and ultimate strength but lower Young’s modulus compared to their solvent-cast counterparts. In PLA/PCL blends, electrospinning enhanced elongation at break without significant stiffness loss. However, PLA/PEO blends showed improved hydrophilicity at the cost of reduced mechanical properties, regardless of processing method. In summary, electrospun films consistently displayed superior flexibility and mechanical integrity compared to their solvent-cast counterparts, primarily due to their fibrous structure, suppressed phase separation, and low crystallinity.

The use of the DCM/DMF mixture in solvent casting induced phase separation, severely compromising the mechanical performance of the PLA films. The inherent immiscibility of PLA with PCL and PEO further intensified this effect in solvent-cast blends. In contrast, electrospinning suppressed phase separation in PLA/PCL blends, enhancing their flexibility while maintaining mechanical integrity.

Surface properties also varied with processing; electrospun neat PLA and PLA/PCL blends were more hydrophobic than their solvent-cast counterparts, while both electrospun and solvent-cast PLA/PEO blends exhibited significantly enhanced hydrophilicity.

The selection of blend components was crucial in determining the material properties of the films (Table S2). In electrospun films, PCL effectively enhanced flexibility while maintaining mechanical integrity, creating homogeneous blends with controlled phase separation. This makes electrospun PLA/PCL blends well-suited for applications requiring improved toughness. In contrast, PEO significantly increased hydrophilicity but compromised mechanical performance due to pronounced phase separation, particularly in solvent-cast films. However, despite PEO’s inherent immiscibility with PLA and incompatibility in the solvent mixture, electrospinning successfully enabled the fabrication of phase-separated PLA/PEO blends with retained mechanical integrity. As a result, electrospun PLA/PEO blends are suitable for applications prioritizing surface wettability, such as drug delivery systems or hydrophilic coatings.

Overall, this study underscores electrospinning as a superior processing method for optimizing the mechanical and surface properties of thin films from PLA blends. These insights provide guidelines for designing tailored PLA-based materials, particularly for applications requiring a balance of flexibility, mechanical integrity, and surface functionality.

## Figures and Tables

**Table 1:** The solubility of PLA, PCL, and PEO in DCM, DMF, and the DCM/DMF mixture.

	DCM		DMF		DCM/DMF (7:3 *v*/*v*)	
	g solute/100 g solvent	*w*/*v* %	g solute/100 g solvent	*w*/*v* %	g solute/100 g solvent	*w*/*v* %
PLA	55.10	73.28	3.01	2.84	27.74	33.68
PCL	40.10	53.33	24.83	23.44	35.52	43.09
PEO	negligible		negligible		negligible	

**Table 2:** Melting temperatures and crystallinity of PLA, PCL, and PEO in the electrospun and solvent-cast films of neat PLA, neat PCL, PLA/PCL blends, PLA/PEO blends, and pristine PEO.

		$T_{m,PLA}$ (°C)	${∆H}_{cc,PLA}$ (J/g)	${∆H}_{m,PLA}$ (J/g)	$X_{c,PLA}$ (%)	$T_{m,PCL}$ (°C)	${∆H}_{m,PCL}$ (J/g)	$X_{c,PCL}$ (%)	$T_{m,PEO}$ (°C)	${∆H}_{m,PEO}$ (J/g)	$X_{c,PEO}$ (%)
PLA	ES	167.3	24.93	52.84	30.0	-	-	-		-	-
	SC	169.2	0	67.33	72.4	-	-	-	-	-	-
PLA/PCL (1:1)	ES	166.9	17.16	54.64	40.3	55.4	60.91	43.7	-	-	-
	SC	168.9	0	64.38	69.2	61.0	94.96	68.1	-	-	-
PLA/PCL (1:2)	ES	167.1	17.97	59.64	44.8	55.7	63.93	45.8	-	-	-
	SC	167.4	0	61.92	66.6	60.6	94.65	67.9	-	-	-
PCL	ES	-	-	-	-	55.9	71.49	51.3	-	-	-
	SC	-	-	-	-	59.8	98.71	70.8	-	-	-
PLA/PEO (1:1)	ES	167.7	4.50	57.26	56.7		-	-	60.6	156.44	73.3
	SC	168.5	0	64.68	69.6		-	-	61.9	200.6	94.0
PLA/PEO (1:2)	ES	166.9	4.89	57.75	56.8		-	-	60.9	170.55	79.9
	SC	168.3	0	64.17	69.0		-	-	62.8	201.4	94.4
PEO	pristine	-	-	-	-	-	-	-	62.2	205.9	96.5

**Table 3:** A comparison of mechanical properties of the electrospun and solvent-cast films.

		Young’s Modulus	Ultimate Strength	Elongation at Break	Key Observations
PLA	ES	Lower	Higher	Greater	Flexible, lower crystallinity; aligned nanostructure enhances elongation.
	SC	Higher	Lower	Brittle	High crystallinity leads to stiffness but causes brittleness.
PLA/PCL (1:1)	ES	Comparable to PLA	Could not be determined	>80% (estimated)	PCL increases flexibility; phase separation is suppressed, maintaining ductility.
	SC	Lower than PLA	Reduced	Moderate	Large phase separation leads to weak interfacial adhesion, reducing strength.
PLA/PCL (1:2)	ES	Comparable to PLA	Could not be determined	>80% (estimated)	Higher PCL content further enhances flexibility and fiber uniformity.
	SC	Further reduced	Moderate	Increased	Heterogeneous phase morphology lowers stiffness but improves elongation.
PLA/PEO (1:1)	ES	Lower than PLA	Reduced	Lower	PEO increases hydrophilicity but weakens mechanical strength.
	SC	Fragile	Not measurable	Not measurable	Large-scale phase separation.
PLA/PEO (1:2)	ES	Lower than PLA	Higher than 1:1	Lower	Higher PEO content further reduces mechanical integrity.
	SC	Fragile	Not measurable	Not measurable	Excessive phase separation.

## Data Availability

The data presented in this study are available on request from the corresponding author.

## Supplementary Materials

The following supporting information can be downloaded at: https://www.mdpi.com/article/10.3390/polym17060800/s1, Figure S1: ATR-FTIR spectra of electrospun PCL films, solvent-cast PCL films, and pristine PEO; Figure S2: XRD patterns of (a) the electrospun and solvent-cast PCL films and (b) the pristine PEO; Figure S3: DSC thermograms of (a) the electrospun and solvent-cast PCL films and (b) the pristine PEO; Figure S4: Representative stress-stretch curves of the electrospun and solvent-cast films of neat PCL; Figure S5: SEM images showing the fracture surface of solvent-cast PLA films prepared from a PLA solution in which DCM served as the sole solvent (without the addition of DMF); Figure S6: AFM images of a single electrospun PCL fiber; Figure S7: Representative photomicrographs of the water contact angles of the electrospun and solvent-cast films of neat PCL; Table S1: Morphological features of the electrospun and solvent-cast PLA-based films from SEM and AFM analysis; Table S2: Comparative analysis of the effects of PCL and PEO additives on PLA-based blends across key material properties and processing challenges.
