# Supplementary material for: Enhancing the Flexibility and Hydrophilicity of PLA via Polymer Blends: Electrospinning vs. Solvent Casting
Source: Polymers (Basel). 2025 Mar 18;17(6):800. doi: 10.3390/polym17060800 (PMC11945453; doi:10.3390/polym17060800)
Supplement: Supplementary file 1 [file polymers-17-00800-s001.zip › polymers-3517372-supplementary.pdf]

Supporting Information

Enhancing the Flexibility and Hydrophilicity of PLA via Polymer Blends: Electrospinning vs. Solvent Casting

Qi-Hong Weng [1,†], Ming-Hsien Hu [2,3,†], Ji-Feng Wang [1,†] and Jin-Jia Hu [1,*]

[1] Department of Mechanical Engineering, National Yang Ming Chiao Tung University, Hsinchu 300, Taiwan; a0953661618@gmail.com (Q.-H.W.); www.806640420@gmail.com (J.-F.W.); jjhu@nycu.edu.tw (J.-J.H.)
[2] Department of Post-Baccalaureate Medicine, National Chung Hsing University, Taichung 402, Taiwan; minghsienhu@nchu.edu.tw
[3] Orthopedic Department, Showchwan Memorial Hospital, Changhua 500, Taiwan; minghsienhu@gmail.com
* Correspondence: jjhu@nycu.edu.tw
† These authors contributed equally to this work.

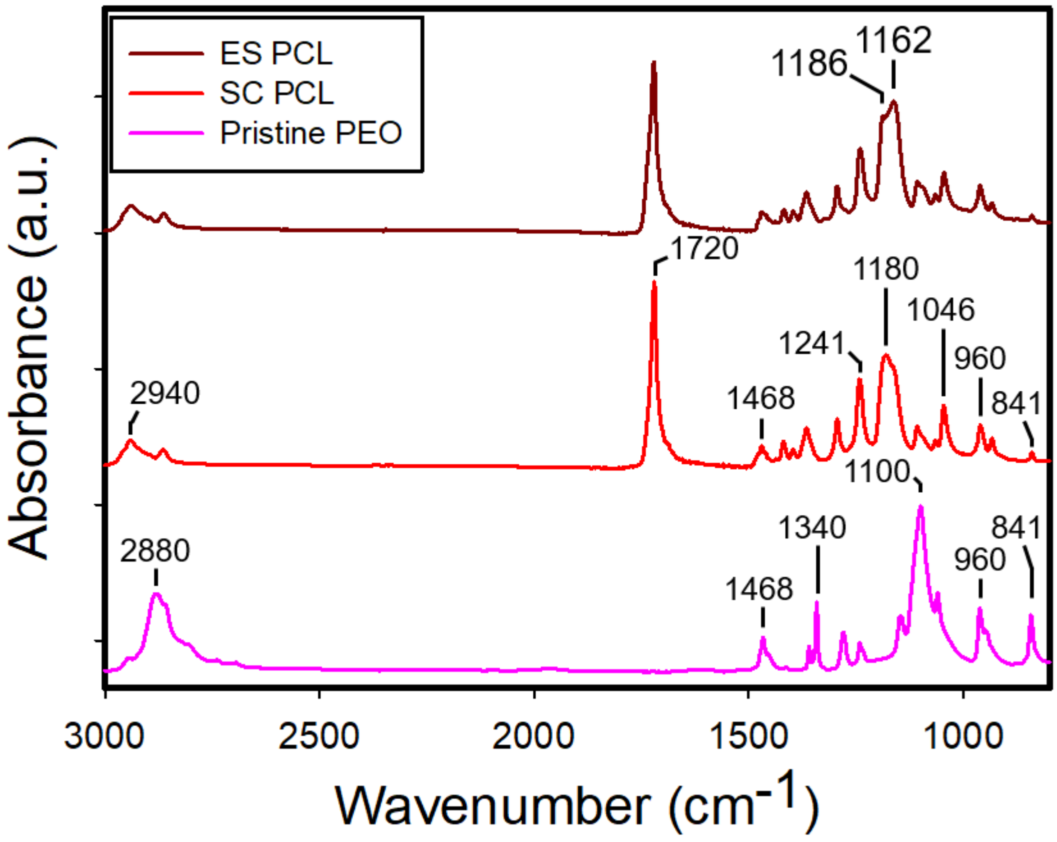

Figure S1. ATR-FTIR spectra of electrospun PCL films, solvent-cast PCL films, and pristine PEO.

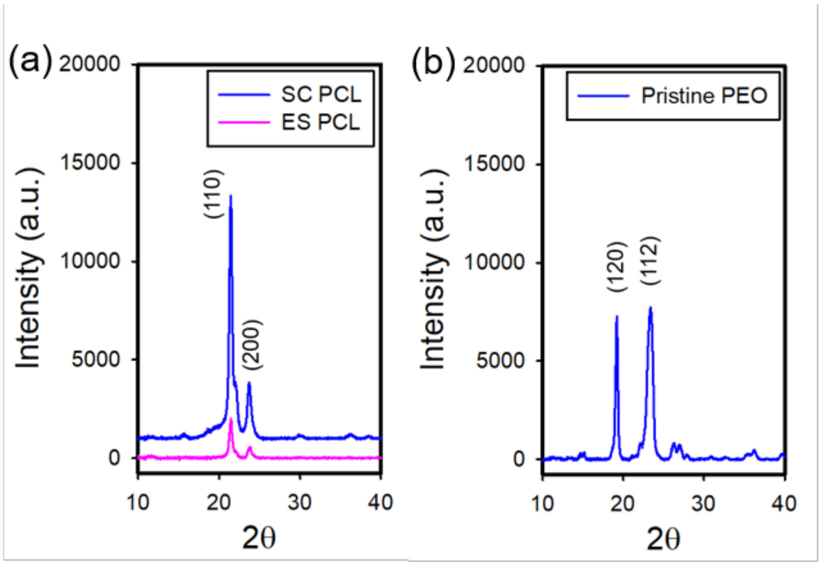

Figure S2. XRD patterns of (a) the electrospun and solvent-cast PCL films and (b) the pristine PEO.

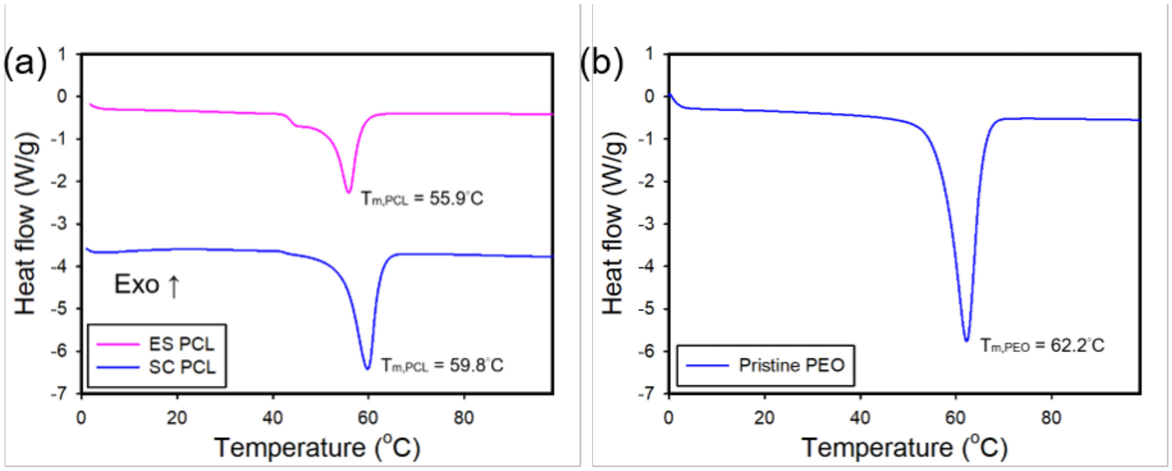

Figure S3. DSC thermograms of (a) the electrospun and solvent-cast PCL films and (b) the pristine PEO.

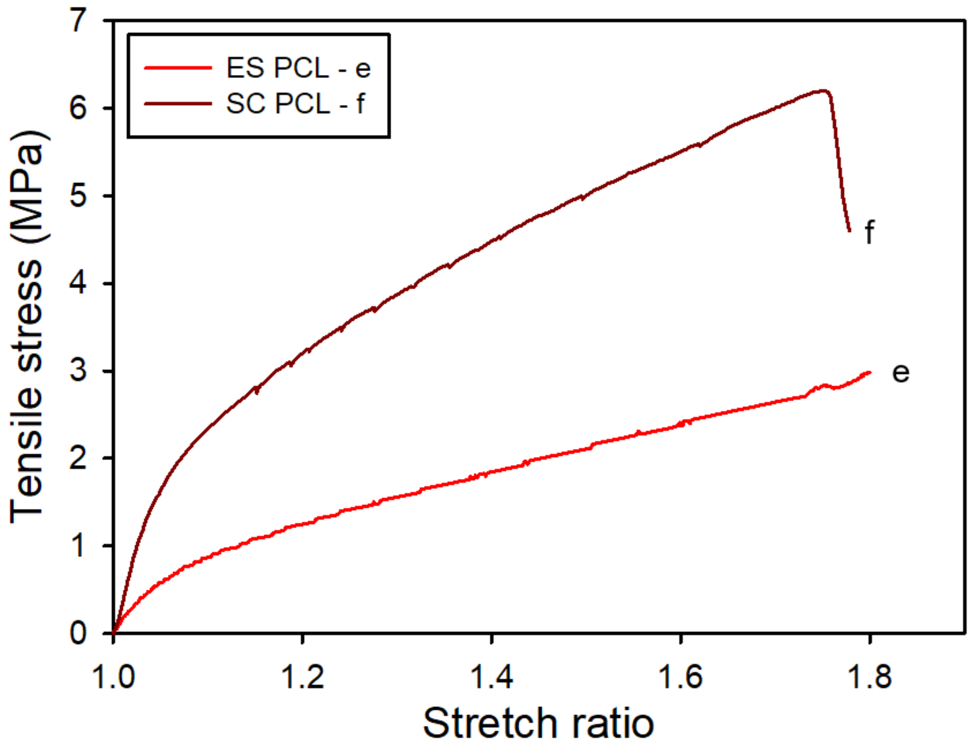

Figure S4. Representative stress-stretch curves of the electrospun and solvent-cast films of neat PCL.

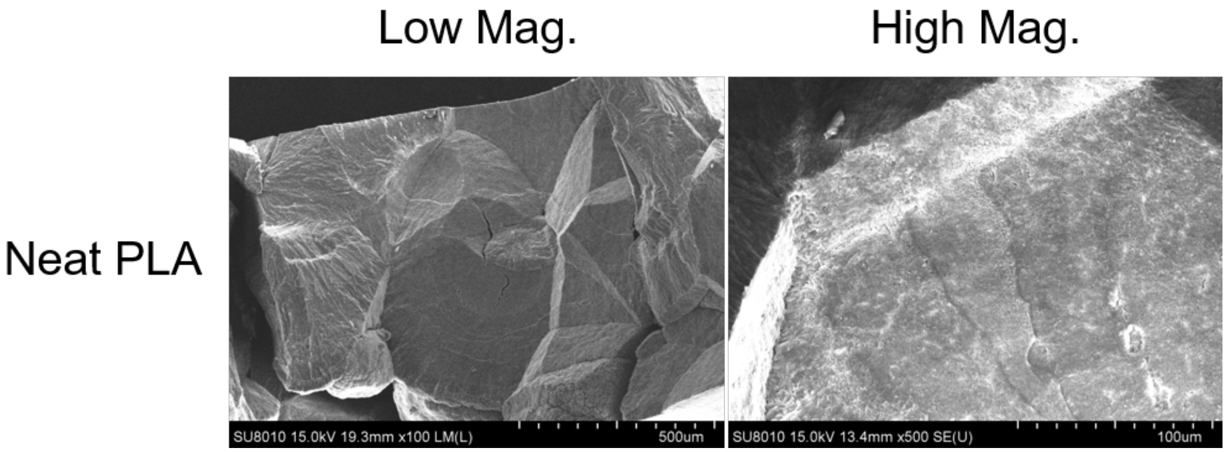

Figure S5. SEM images showing the fracture surface of solvent-cast PLA films prepared from a PLA solution in which DCM served as the sole solvent (without the addition of DMF).

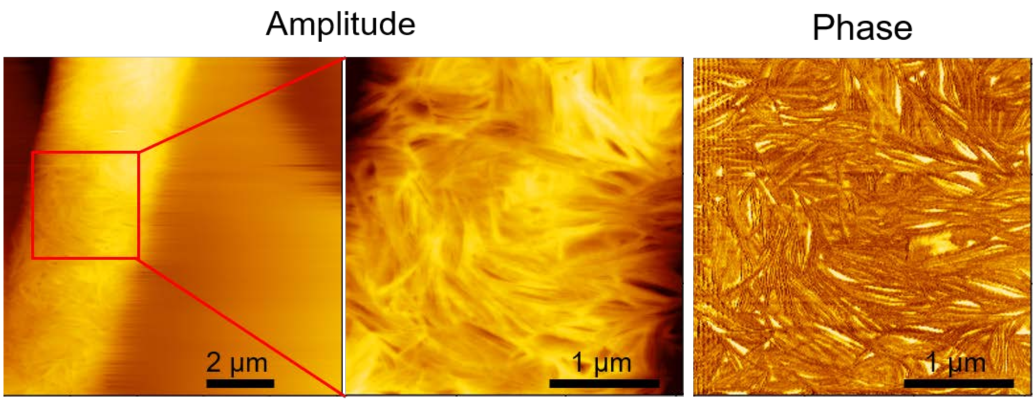

Figure S6. AFM images of a single electrospun PCL fiber.

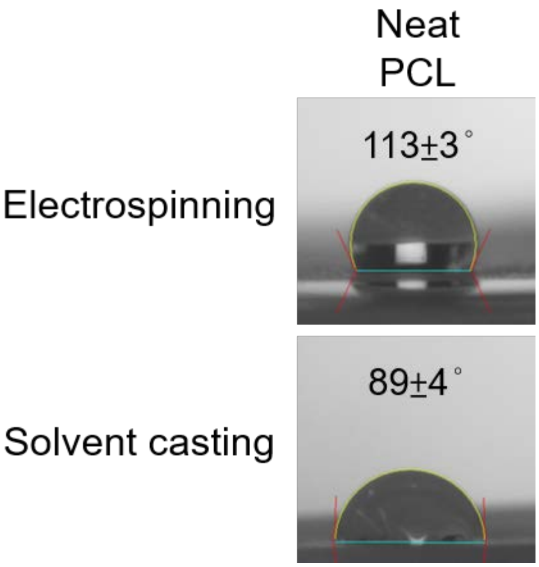

Figure S7. Representative photomicrographs of the water contact angles of the electrospun and solvent-cast films of neat PCL.

Table S1. Morphological features of the electrospun and solvent-cast PLA-based films from SEM and AFM analysis.

		Fiber/Film Morphology	Phase separation (SEM)	Surface Features (AFM)
**PLA**	**ES**	Smooth, uniform fibers	No visible phase separation	Aligned crystalline domains parallel to fiber axis
	**SC**	Smooth film with fine micro-textures	Non-solvent induced phase separation	–
**PLA/PCL (1:1)**	**ES**	Fibers with slight diameter variation	Reduced phase separation compared to solvent casting	Irregularly distributed domains on the fiber
	**SC**	Film with spherical PLA-rich domains in a continuous PCL-rich matrix	Significant phase separation	–
**PLA/PCL (1:2)**	**ES**	More uniform fibers	Phase separation still suppressed	–
	**SC**	Highly porous interconnected morphology	PCL-rich continuous phase, PLA dispersed	–
**PLA/PEO (1:1)**	**ES**	Thick fibers with minor texturing	Phase separation visible	Pebble-like nanoscale domains aligned along fiber axis
	**SC**	Snowflake-like crystalline structures on surface	Macroscopic phase separation	–
**PLA/PEO (1:2)**	**ES**	Thickest fibers with rough surface	Phase separation evident	–
	**SC**	Porous, irregular morphology	PEO crystalline domains dispersed in PLA matrix	–

Table S2. Comparative analysis of the effects of PCL and PEO additives on PLA-based blends across key material properties and processing challenges.

Metric	PLA + PCL	PLA + PEO	Conclusion
**Mechanical Flexibility**	Significant improvement with PCL addition, especially for electrospun films, which show high elongation at break.	Reduced flexibility and strength due to phase separation in electrospun films.	PCL enhances flexibility, while PEO compromises mechanical performance.
**Young's Modulus**	Similar to neat PLA in electrospun films but reduced in solvent-cast films.	Significantly lower than neat PLA in electrospun films.	PCL blends maintain stiffness better than PEO blends.
**Crystallinity**	Lower in electrospun films compared to solvent-cast films; increases with PCL content.	High crystallinity in solvent-cast films; lower crystallinity in electrospun films.	PCL enables controlled crystallinity; PEO drastically increases crystallinity in solvent casting.
**Surface Morphology**	Smooth fibers with refined phase separation in electrospinning; irregular and porous in solvent casting.	Phase-separated domains with rough, pebble-like morphology in electrospinning.	PCL creates more homogeneous and aligned structures compared to PEO.
**Hydrophilicity**	No significant change from neat PLA.	Markedly increased hydrophilicity, especially in solvent-cast films.	PEO dramatically improves hydrophilicity at the expense of mechanical performance.
**Thermal Properties**	Distinct melting peaks for PLA and PCL.	Distinct melting peaks for PLA and PEO.	PCL maintains distinct thermal properties, while PEO shows less compatibility.
**Processing Challenges**	Better apparent miscibility in electrospinning.	Pronounced phase separation; difficult to fabricate intact solvent-cast films.	PCL is easier to process with PLA compared to PEO.
